# Aerial Grasping with a Lightweight Manipulator Based on Multi-Objective Optimization and Visual Compensation

**DOI:** 10.3390/s19194253

**Published:** 2019-09-30

**Authors:** Haoyao Chen, Fengyu Quan, Linxu Fang, Shiwu Zhang

**Affiliations:** 1 School of Mechanical Engineering and Automation, Harbin Institute of Technology (Shenzhen), Shenzhen 518055, China; qfyhit@foxmail.com (F.Q.); flxu2002@gmail.com (L.F.); 2 Department of Precision Machinery and Precision Instrumentation, University of Science and Technology of China, Hefei 230022, China; swzhang@ustc.edu.cn

**Keywords:** visual tracking, visual compensation, trajectory planning, aerial manipulation

## Abstract

Autonomous grasping with an aerial manipulator in the applications of aerial transportation and manipulation is still a challenging problem because of the complex kinematics/dynamics and motion constraints of the coupled rotors-manipulator system. The paper develops a novel aerial manipulation system with a lightweight manipulator, an X8 coaxial octocopter and onboard visual tracking system. To implement autonomous grasping control, we develop a novel and efficient approach that includes trajectory planning, visual trajectory tracking and kinematic compensation. Trajectory planning for aerial grasping control is formulated as a multi-objective optimization problem, while motion constraints and collision avoidance are considered in the optimization. A genetic method is applied to obtain the optimal solution. A kinematic compensation-based visual trajectory tracking is introduced to address the coupled affection between the manipulator and octocopter, with the advantage of discarding the complex dynamic parameter calibration. Finally, several experiments are performed to verify the effectiveness of the proposed approach.

## 1. Introduction

Unmanned aerial vehicle (UAV) that is equipped with a manipulator, namely unmanned aerial manipulator (UAM), is a popular research topic because of its immense potential for various applications, including express transportation, construction and maintenance, and manipulations in dangerous places that are difficult to reach by humans or ground mobile robots. Although UAVs have been well studied [1], UAMs still present significant challenges in perception and control, mainly because of the considerably complex kinematics/dynamics and motion constraints of the coupled UAV-manipulator system.

Many researchers have proposed interesting studies on aerial transportation and manipulation systems, including the mechanical and controller design of cable suspended systems and aerial grippers [2]. Like a tower crane system, UAV lifting a load with a cable-suspended device is a beneficial solution for aerial transportation [3,4,5,6]. Although cable-suspended systems is able to provide high maneuverability for load transportation on all terrains, these systems are limited in the application of aerial manipulation, such as grasping. A textbook containing the latest research results about the cable-suspended UAVs is provided in [7]. To achieve automatic aerial object gripping and transportation, various task-adaptive grippers or end-effectors directly attached to the UAV base have been proposed in the literature [8,9,10]. These grippers are directly equipped on the UAV body base and thus can be easily controlled like a nominal UAV. However, they lack sufficient degree-of-freedom (DoF) to implement complex manipulations.

Compared with the aforementioned systems, UAV equipped with a flexible multi-DoF manipulator is able to perform much more flexible manipulations even under substantially complex environments [11]. However, the UAMs systems suffer from more challenging problems in perception, trajectory planning and manipulation control. Several studies have been performed on UAMs’ trajectory planning and manipulation control [12,13,14,15,16,17]. The control of UAM in the existing approaches requires the precise modeling of dynamics [11]. However, dynamics modeling is extremely challenging because many physical parameters, such as rotational inertia [18] or external disturbances, are difficult to measure accurately and the dynamic parameters often vary gradually and even substantially during the operation. In addition, the adaptive solutions for dynamic control need sufficient excitation to prevent divergence. These problems lead to the inconvenience and poor performance of practical applications. Furthermore, most of the relevant studies on UAM are based on simulation or do not consider the control constraints, such as collision avoidance and motor limitations, under practical environments. Particularly, the existing visual servoing approaches have difficulty in completing complex collision-free grasping tasks.

This paper aims to develop a novel approach to address the aforementioned problems. The approach comprises visual target detection, trajectory generation, and visual trajectory tracking control. It implements real-time autonomous grasping of a target with a lightweight aerial manipulator. All algorithms are executed without off-board computation assistance. The contributions are threefold.

Firstly, to reduce dynamic interference, an UAM with a lightweight manipulator is designed, and the kinematic model and visual observation model are deduced. The system mainly comprises a 4-DoF lightweight manipulator, a coaxial octocopter, a camera sub-system and onboard processing modules. The camera sub-system provides real-time target detection. The target detection, together with the UAV and manipulator control algorithms, are performed onboard. The investigation of system models lays a foundation to the follow-on algorithm development of autonomous trajectory generation and visual grasping control. The designed UAM realizes anti-interference ability against not only dynamic coupling between manipulator and UAV body but also the aerodynamic disturbance. Compared the existing solutions for anti-interference, our system does not need dynamic controllers, and thus it is easily integrated with commercial onboard autopilots and computation units. Experimental results were provided to illustrate the performance.

Secondly, a trajectory planning algorithm for aerial grasping is developed based on multi-objective optimization formulation; it guarantees the grasping success and safety. Grasping or other manipulations require UAM body to avoid collision with obstacles as well as satisfying the mechanical and electrical limitations. The existing approaches [11] for the trajectory planning of aerial grasping generally seek a feasible solution in open areas without considering environmental obstacles and constraints. They also ignored the factor that the grasping should be immediately completed and the moving distance of the end-effector should also be as short as possible because of UAV’s limited power capability. By considering all the aforementioned factors, the trajectory planning problem is formulated as an optimization problem and the genetic optimizer nondominated sorting genetic algorithm II (NSGA-II) [19] is utilized to obtain a feasible solution. Although the optimization-based solution is not a novel idea in ground manipulator and other mobile robotic fields, its application in an aerial system is still a challenging problem. The planning and control of aerial grasping need to address many problems like the coupling effect, aerial model, motion constraints, real-time performance and aerial disturbance; these problems are generally not existing in the ground robots.

Thirdly, a trajectory-tracking controller based on real-time visual compensation is developed; the aerodynamic disturbance and UAV’s coupling effect on the pose control of the end-effector are reduced. The movement of the manipulator or other unstable factors, such as wind disturbance, may change the UAV body pose or target object pose during the tracking process. The influence of dynamic and environmental disturbances in trajectory grasping presents difficulty in ensuring successful grasping. Many existing approaches are based on image-based visual servoing technology where the camera was installed at the end effector of the robotic arm, and therefore may partially reduce the affection of disturbance; however, the approaches suffer from loss of view when the camera is too close to the target. The proposed solution reduces the influence based on real-time compensation-based feedback control with visual target information. The experimental results show that the controller enables the aerial manipulator to grasp the target object successfully even when the position of the aerial manipulator or target object considerably varies.

## 2. Related Work

UAMs have immense potential applications, and the problems of trajectory planning and manipulation control of UAM have attracting increasing attention in robotic fields.

Mebarki et al. [12] designed an image-based kinematic visual servoing controller to generate trajectory tracked by a low-level dynamic controller. The joint limits of the UAM system were considered in the approach. The null space-based behavioral (NSB) control method derived from manipulator control was utilized to achieve a secondary task. However, no obstacle avoidance was considered in the trajectory planning, and only simulations were performed. Kim et al. [17] also developed a similar two-layer controller with [12]; the main difference lies in that a passivity-based adaptive controller based on dynamic model was proposed for the combined UAM system. Although the self-body contact avoidance was taken into account, the obstacle avoidance was overlooked in the trajectory generation. In addition, the camera was installed at the end effector of the robotic arm, and therefore the captured image varies with the motions of the robotic arm as well as the UAV.

Lippiello et al. [13] further extended the NSB based work and developed a hierarchical task-composition control framework for aerial manipulation; several tasks including gripper pose tracking, joint limits avoidance, field of view (FOV) of the camera, etc., were formulated. Similarly, Baizid et al. [20] presented a control framework based on NSB to address the cross-coupling effect between the manipulator and UAV. Furthermore, Muscio et al. [21] proposed a three-layer control architecture for coordinated formation control of multiple UAMs. The centralized top layer plans desired trajectories for each UAM’s end-effector; the NSB method is applied to generate UAM’s motion references for the bottom dynamic controller. Similar to the work [13], the task priority was also employed in [22,23] to generate trajectories for cooperative transportation using multiple aerial manipulators. The dynamic movement primitives (DMPs) were utilized to realize obstacle avoidance in unknown environments. A sliding adaptive controller was proposed to compensate the dynamic uncertainties. Although obstacle avoidance can be achieved with the behavioral or task-based framework, it generally only benefits for manipulators with high degree-of-freedom and fails to guarantee the simultaneous success of all executed tasks.

Despite of NSB methodology, some other interesting approaches have also been developed in the literature. Seo et al. [24] developed a stochastic model predictive control (MPC) framework, and the aerial grasping control was implemented by minimizing the feature tracking errors and control inputs. However, it is complex to consider the avoidance of UAM constraints and environmental obstacles into the MPC framework. Garimella et al. [16] presented a nonlinear MPC method based on multi-body system dynamics and achieved optimized performance. However, the approach overlooked the collision avoidance in the multi-body system and between system and environmental obstacles. Thomas et al. [25] developed an UAM equipped with a monocular camera, and formulated the dynamics directly in the virtual image plane. By modeling the UAM as a differentially-flat system and servoing the image features as flat outputs, a trajectory generation approach for agile grasping was proposed directly in the image feature space by planning the trajectories of image features. Seo et al. [26] formulated the trajectory planning as a sequential quadratic programming problem and the planning is performed on selected flat outputs by utilizing the differential flatness advantage of multirotors. The collision between the multirotor base (or the end-effector of the robotic arm) and environmental obstacles were taken into account in the planning. However, the joint limits and manipulator collision constraint were not considered in their approach.

Some well-known approaches used in mobile robots, like rapidly exploring random tree (RRT) and DMPs, have been applied to aerial manipulation. Lee et al. [27] developed a trajectory planning algorithm for cooperative aerial transportation by exploiting RRT* and DMPs. The RRT* was utilized to generate the desired trajectory for each aerial manipulator handling environmental obstacles, while the DMPs was utilized to modify the RRT-based trajectory to avoid unknown obstacles. It only considers 2D spaces in the horizontal plane for each aerial manipulator. Kim et al. [28] further utilized Parametric Dynamic Movement Primitives (PDMPs) to learn scalable control policies from multiple demonstrations, and utilized Gaussian Process Regression (GPR) to acquire style parameters of PDMPs according to the environmental parameters. Tognon et al. [29] utilized the RRT algorithm for generating a trajectory on task space but not the full state space, and they developed a control strategy including dynamic and inverse kinematics controllers as a steering method for RRT’s extension. This RRT-based kinodynamic planning approach can achieve the validation of robotic and environmental constraints; however, it also suffers from the computation problem and is unable to obtain optimal solution. Simulations were performed to illustrate the performance. The RRT-based approach cannot give deterministic safety guarantee or optimality.

There is also a lot of research focus on the dynamic control problem of aerial manipulators; many of them only provide simulations. The trajectory generation problem are overlooked in these research. Lippiello et al. [30] developed a cascade control structure with an inner loop for inverse dynamic motion control and an external loop implementing visual-impedance control. The approach provides a reference trajectory for the inner loop. Simulations were presented. Kim et al. [31] utilized feedback linearization to realize dynamic regulation control for a UAM where a heavy manipulator is mounted far from the center of mass (CoM) of UAM body and is able to move in 3D. Simulations were presented. Heredia et al. [15] presented two separate controllers for the UAV and manipulator arm, i.e., a backstepping-based controller for UAV that considers the full dynamics of the 7-DoF manipulator arm and an admittance controller for manipulator arm. Trajectory planning and obstacle avoidance were overlooked in the approach. Kim et al. [32] utilized disturbance observer (DOB)-based approach to recover the dynamics of a multirotor combined with additional objects and then control the complex system similar to the bare multirotor.

## 3. Problem Formulation

**Figure 1:**
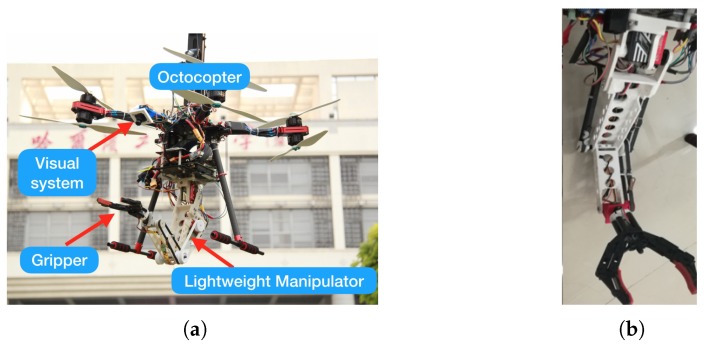
The proposed aerial manipulator system. (**a**) modules; (**b**) 4-DoF manipulator with a gripper.

This study aims to develop an aerial manipulator system with a lightweight manipulator for autonomous target grasping. The aerial manipulator system comprises a 4-DoF lightweight manipulator, a coaxial octocopter, a camera sub-system, and onboard processing modules (see Figure 1a). The camera sub-system, consisting of a monocular camera and a 1-DoF pitch servo motor, is developed to track the target for target grasping. The camera rotates in the pitching direction driven by the servo motor. The pitching DoF and the mobility of UAV in the yaw direction ensure that the target is not easily lost in the field of view of the camera during the task. Note the proposed aerial manipulator can not only complete object grasping but also be easily adaptable for other related manipulations, such as visual surveillance, tightening or loosening screws, placing objects or knocking off objects.

Inspired from the manipulator in [33], we design a lightweight manipulator by arranging the power drive unit (i.e., motors) on the base of the arm to reduce the instability of the system dynamics during the manipulator movement. Different from [33], we replace the complex 2-DoF differential mechanism by a high-torque servo motor, and the maneuverability of UAV is utilized to implement the other necessary DoFs; the modified mechanism is much simpler and easier to maintain, as well as satisfying the manipulation requirements. The developed X8 coaxial octocopter provides sufficient payload for the manipulator and target. The mechanical gripper, as shown in Figure 1b, is easily replaced by other gripper types, such as the lightweight shape memory alloy-based gripper [34] developed in our group.

The 4-DoF octocopter and 4-DoF manipulator provide an 8-DoF movement. Therefore, the system enables the end-effector to achieve a 6-DoF movement and provides additional movement DoFs for the collision avoidance of UAM. However, the system control is complex and challenging because DoFs are considerably coupled. That is, the motion of the manipulator will affect the hovering stability of the octocopter, while the octocopter influences the control of the manipulator. Additionally, the system control should address a few constraints, such as joint limits, UAV velocity and joint velocity limits, and collision avoidance. Furthermore, the end-effector moving distance and action time duration should be considerably limited to save energy. Generally, the aforementioned constraints and objectives are impossible to satisfy simultaneously. Therefore, a cost function that balances the aforementioned constraints and objectives should be formulated to generate a trajectory for successfully implementing the autonomous grasping task.

To address the mentioned problems, a novel vision-based approach is proposed including three stages, namely, multi-objective optimization-based trajectory generation, visual motion compensation, and trajectory tracking control. Without the need to calibrate the dynamic parameters or use adaptive control scheme, the developed system based on kinematic model is easily implemented with commercial autopilot and onboard computation unit. The kinematics model as well as the visual observation model of the UAV-manipulator system are firstly built. The camera subsystem provides the target location for UAV to approach and grasp the target. Once the aerial manipulator reaches the desired location, trajectory planning with multi-objective optimization is performed to generate the trajectory in the manipulator’s joint configuration space. Thereafter, the trajectory is corrected in real time with the visual feedback and the manipulator performs target grasping along the compensated trajectory. The aerodynamic disturbance and the coupled affection between the manipulator and UAV is released based on the compensation-based trajectory correction.

## 4. Modeling of the UAV-Manipulator System

**Figure 2:**
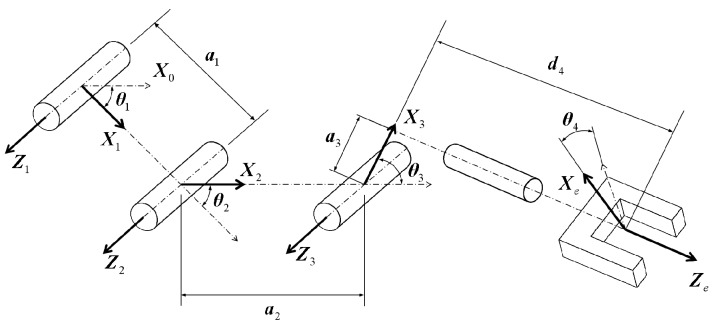
Coordinate frames of the proposed manipulator.

Figure 2 illustrates the relationship between the coordinate frames of the manipulator joints and the proposed 4-DoF manipulator. Table 1 shows the Denavit–Hartenberg parameters. The following notations are firstly introduced before presenting the modeling. Let *b* denote the UAV body frame, and *e* denote the end-effector frame (i.e., 4th link frame). Let indexes 0, 1, 2, and 3 denote the base frame and the 1st, 2nd, and 3rd link frames, respectively. Matrix $R_{k}^{i}\in SO\left(3\right)$ denotes the rotation transformation of frame *k* from frame *i* , and vector $t_{k}^{i}\in R^{3}$ denotes the translation of frame *k* from frame *i*. $T_{k}^{i}=\left[R_{k}^{i}\;t_{k}^{i};\;0_{3}^{T}\;1\right]\in SE\left(3\right)$ denotes the transformation of frame *k* from frame *i*. For example, $T_{0}^{b}$ denotes the transformation of the manipulator base from the UAV body. The transformation of the end-effector from the manipulator base is expressed as follows:

(1) $$T_{e}^{0}=T_{1}^{0}T_{2}^{1}T_{3}^{2}T_{e}^{3}=\left[\begin{array}{cc} R_{e}^{0} & t_{e}^{0}\\ 0_{3}^{T} & 1 \end{array}\right].$$

The forward kinematics of the aerial manipulator is defined as

(2) $$T_{i}^{b}=T_{0}^{b}T_{i}^{0},$$

where i∈(1,2,3,e) indexes the *i*th link frame. In addition, the detailed expression of the transformation matrices can be found in the literature. Therefore, the Cartesian coordinates of the *i*-th joint w.r.t. the body frame is given as

(3) $$p_{i}={\left(T_{i}^{b}\left(1,4\right),T_{i}^{b}\left(2,4\right),T_{i}^{b}\left(3,4\right)\right)}^{T},$$

where $T_{i}^{b}\left(k,l\right)$ denotes the element at row *k* and column *l* of matrix $T_{i}^{b}$. To deduce the inverse kinematics, i.e., calculating the joint angles $\left(\theta_{1},\theta_{2},\theta_{3},\theta_{4}\right)$ from the a priori known $T_{e}^{0}$, we rewrite matrix $T_{e}^{0}$ as

(4) $$T_{e}^{0}=\left[\begin{array}{cccc} n_{x} & o_{x} & a_{x} & p_{x}\\ n_{y} & o_{y} & a_{y} & p_{y}\\ n_{z} & o_{z} & a_{z} & p_{z}\\ 0 & 0 & 0 & 1 \end{array}\right].$$

Considering Equations (1) and (4), we have

(5) $$\left\{\begin{array}{c} \theta_{4}=arctan\left(n_{z}/o_{z}\right),\\ \theta_{1}+\theta_{2}+\theta_{3}=arctan\left(-a_{z}/a_{y}\right),\\ p_{z}=0, \end{array}\right.$$

where $p_{z}=0$ because all joints of the manipulator are designed in a common plane as shown in Figure 1b. Solving Equations (1), (4) and (5), we then have

(6) $$\left\{\begin{array}{c} \theta_{1}+\theta_{2}=-2arctan\left(\left(s-2a_{2}m\right)/e_{2}\right),\\ \theta_{1}=2arctan\left(\left(s+2a_{1}m\right)/e_{1}\right) \end{array}\right.$$

or

(7) $$\left\{\begin{array}{c} \theta_{1}+\theta_{2}=-2arctan\left(\left(-s-2a_{2}m\right)/e_{2}\right),\\ \theta_{1}=2arctan\left(\left(-s+2a_{1}m\right)/e_{1}\right) \end{array}\right.$$

where

(8) $$\left\{\begin{array}{c} s=\sqrt{s_{1}\times s_{2}},\\ s_{1}=m^{2}+n^{2}-a_{1}^{2}+2a_{1}a_{2}-a_{2}^{2},\\ s_{2}=-m^{2}-n^{2}+a_{1}^{2}+2a_{1}a_{2}+a_{2}^{2},\\ e_{1}=m^{2}+n^{2}+a_{1}^{2}+2a_{1}n-a_{2}^{2},\\ e_{2}=m^{2}+n^{2}-a_{1}^{2}+2a_{2}n+a_{2}^{2},\\ m=p_{y}-a_{3}s_{123}+d_{4}c_{123},\\ n=p_{x}-a_{3}c_{123}-d_{4}s_{123}, \end{array}\right.$$

where $s_{123}$ and $c_{123}$ are the abbreviations of $sin(\theta_{1}+\theta_{2}+\theta_{3})$ and $cos(\theta_{1}+\theta_{2}+\theta_{3})$, respectively. The two solutions of $\theta_{1}$ and $\theta_{2}$ are obtained from Equations (6) and (7). Thereafter, two sets of joint angles $\theta_{i}\left(i=1,2,3,4\right)$ are obtained by substituting the two solutions into Equation (5). Each solution represents a continuous work space; however, the invalid solution is rejected if it conflicts the geometric constraints. Finally, the inverse kinematics is derived.

Besides the forward and inverse kinematic model, we also need the observation model of the camera system. The camera system in our aerial manipulator is driven with a servo motor, and thus is considered as a 1-DoF robot arm. Note we omit the description of the camera’s projection model for simplicity. Let *a* be the frame of the target object, *c* be the camera frame, and *r* be the camera servo joint frame. The offset transformation $T_{c}^{b}$ from the UAV body to the camera servo joint is directly obtained from the CAD model, while the transformation $T_{c}^{r}$ of the camera frame from the servo motor joint is calibrated by using the motion capture system. The transformation $T_{a}^{b}$ of the target object frame from the UAV body is expressed as follows:

(9) $$T_{a}^{b}=T_{r}^{b}T_{c}^{r}T_{a}^{c}.$$

The value of $T_{a}^{c}$ is obtained once the camera observes the target by using quick response code technology or a stereo or RGB-depth camera. The research of target detection is out of the scope of the paper; we utilize the Apriltag technology [35], a kind of QR code, to obtain $T_{a}^{c}$ directly for simplicity. According to Equation (9), we deduce the observation model where the target’s location w.r.t. the UAV base frame is obtained.

## 5. Trajectory Planning Based on Multi-Objective Optimization

To achieve collision-free grasping or other manipulation tasks in practical environments, a feasible trajectory for the movement of the manipulator should be planned a priori. Additionally, the trajectory should satisfy other non-negligible constraints and objectives. Assume that the UAV body stays at a grasping place where the manipulator has a large redundancy in work space for grasping. In the section, the trajectory of the aerial manipulator is firstly formulated mathematically in the joint configuration space by using quintic curves; each joint’s angle is expressed by a quintic curve. Therefore, the trajectory for each joint has a continuous angle value, angular velocity and angular acceleration. Thereafter, the trajectory planning for aerial grasping is formulated as a multi-objective optimization problem by considering the control and mechanical constraints and objectives. Finally, the efficient optimizer approach NSGA-II is utilized to solve the optimization problem.

### 5.1. Mathematical Trajectory Formulation

The proposed manipulator consists of four joints each driven by a servo motor. Because the wrist joint only changes the end-effector’s attitude but not the position, we only consider the other three DoFs for trajectory planning. Note the proposed approach is also suitable for manipulators with more DoFs. The trajectory planning is performed in the joint configuration space because of the complexity of the Cartesian space. The joints of the aerial manipulator are controlled in the continuous space, thereby enabling the trajectory of each joint to be mathematically described by a continuous curve. The quintic curve is utilized because it has a continuous 2nd-order derivative. The mathematical equation of the *i*th joint angle curve is expressed as

(10) $$\left\{\begin{array}{c} \theta_{i}=c_{i}^{T}{\left[1\;t\;t^{2}\;t^{3}\;t^{4}\;t^{5}\right]}^{T},\\ \omega_{i}=c_{i}^{T}{\left[0\;1\;2t\;3t^{2}\;4t^{3}\;5t^{4}\right]}^{T},\\ \alpha_{i}=c_{i}^{T}{\left[0\;0\;2\;6t\;12t^{2}\;20t^{3}\right]}^{T}, \end{array}\right.$$

where $c_{i}\in R^{6}$ denotes the polynomial parameter vector, *t* denotes the time, and $\omega_{i}$ and $\alpha_{i}$ denote the angular velocity and acceleration, respectively, of the *i*th joint. By stacking all joints together, we have

(11) $$\left\{\begin{array}{c} \Theta =C{\left[1\;t\;t^{2}\;t^{3}\;t^{4}\;t^{5}\right]}^{T},\\ \Omega =\dot{\Theta }=C{\left[0\;1\;2t\;3t^{2}\;4t^{3}\;5t^{4}\right]}^{T},\\ A=\ddot{\Theta }=C{\left[0\;0\;2\;6t\;12t^{2}\;20t^{3}\right]}^{T}, \end{array}\right.$$

where $C\in R^{m\times 6}$ denotes the polynomial parameter matrix, *m* denotes the number of joints involved in the planning and $\Theta \in R^{m}$, $\Omega \in R^{m}$ and $A\in R^{m}$ denote the stacked joint angles, angular velocities and angular accelerations, respectively.

Given the start and goal kinematic information of the manipulator, the quintic curve parameters of the *i*th joint are calculated as follows:

(12) $$c_{i}=\left[\begin{array}{c} \theta_{is}\\ \omega_{is}\\ \frac{\alpha_{is}}{2}\\ \frac{20\theta_{is}-20\theta_{ig}+12t_{g}\omega_{is}+8t_{g}\omega_{ig}+3\alpha_{is}t_{g}^{2}-\alpha_{ig}t_{g}^{2}}{-2t_{g}^{3}}\\ \frac{30\theta_{is}+30\theta_{ig}+16t_{g}\omega_{is}+14t_{g}\omega_{ig}+3\alpha_{is}t_{g}^{2}-2\alpha_{ig}t_{g}^{2}}{-2t_{g}^{4}}\\ \frac{12\theta_{is}-12\theta_{ig}+6t_{g}\omega_{is}+6t_{g}\omega_{ig}+3\alpha_{is}t_{g}^{2}-\alpha_{ig}t_{g}^{2}}{-2t_{g}^{5}} \end{array}\right],$$

where $t_{g}$ denotes the time when the joint reaches the goal angle, $\theta_{is}$ and $\theta_{ig}$ denote the start and goal angles, respectively, of the *i*th joint; $\omega_{is}$ and $\omega_{ig}$ denote the start and goal angular velocities, respectively, and $\alpha_{is}$ and $\alpha_{ig}$ denote the start and goal angular accelerations, respectively.

**Figure 3:**
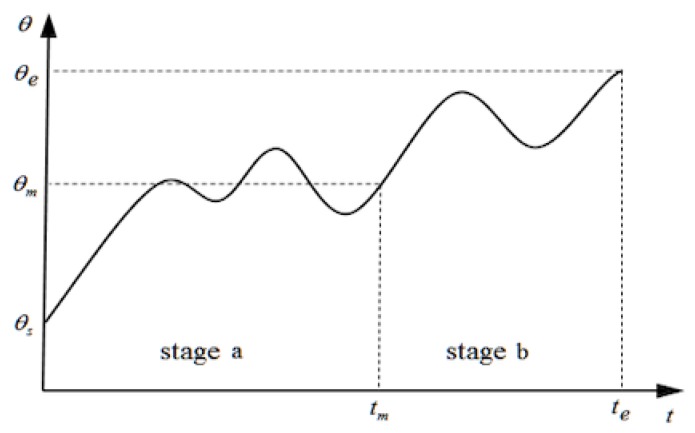
Two-stage quintic curve of a joint angle.

A two-stage quintic curve is utilized to ensure that the trajectory planning has considerable flexibility and exhibits a good computational performance. A demo of the two-stage quintic curve of one joint’s trajectory, denoted as stages *a* and *b*, is shown in Figure 3. Let $C_{a}$ and $C_{b}$ denote the parameter matrix of each stage of the quintic curve. Let $T_{a}$ and $T_{b}$ denote the time durations of the two stages, respectively; we have $T_{a}=t_{m}$ and $T_{b}=t_{g}-t_{m}$, respectively, where $t_{m}$ and $t_{g}$ denote the time at the end of the first and second stages respectively, as shown in Figure 3. From Equation (12), the trajectory curve is determined uniquely with the boundary condition set $B=(t_{m},t_{g},\Theta_{s}^{T},\Omega_{s}^{T},A_{s}^{T},\Theta_{m}^{T},\Omega_{m}^{T},A_{m}^{T},\Theta_{g}^{T},\Omega_{g}^{T},A_{g}^{T})$. $\Theta_{s}$, $\Omega_{s}$, and $A_{s}$ denote the start joint angle, angular velocity and angular acceleration, respectively; $\Theta_{m}$, $\Omega_{m}$, and $A_{m}$ denote the intermediate joint angle, angular velocity and angular acceleration, respectively; $\Theta_{g}$, $\Omega_{g}$, and $A_{g}$ denote the goal joint angle, angular velocity and angular acceleration, respectively.

### 5.2. Objectives and Constraints in Trajectory Planning

The Cartesian coordinates of the *i*th joint calculated from the forward kinematics are obtained from Equation (3). Thereafter, given the joint configuration Θ, we have the Cartesian coordinates of the end-effector w.r.t. of the UAV body as follows:

(13) $$p_{e}=K_{e}\left(\Theta \right)={\left(T_{e}^{b}\left(1,4\right),T_{e}^{b}\left(2,4\right),T_{e}^{b}\left(3,4\right)\right)}^{T},$$

where $T_{e}^{b}$ is a function of Θ. To drive the end-effector to grasp the object as soon as possible, we define the following two objectives:

(14) $$\left\{\begin{array}{c} f_{1}\left(B\right)=t_{g},\\ f_{2}\left(B\right)=L_{arc}, \end{array}\right.$$

where $L_{arc}$ is the trajectory length of the end-effector. To calculate the value of $L_{arc}$, the joint curve with the boundary conditions B is discretized with sampling time Δt . Let $S_{\Theta }$ denote the set of sampled joint angle vectors, $S_{\Omega }$ denote the set of sampled joint angular velocity vectors, and $S_{A}$ denote the set of sampled joint angular acceleration vectors, along the trajectory. The joint angle vector, angular velocity vector and angular acceleration vector obtained at the *j*th sampling stamp are denoted as $\Theta_{j}$ , $\Omega_{j}$ and $A_{j}$ , respectively. The total number of sampling points (i.e., number of vectors in each set) is provided by $N=\frac{t_{g}}{\Delta t}+1$.

The end-effector position at the *j*th sampling stamp, denoted as $p_{e,j}$, is obtained from Equation (13). Let Φ denote the set of $p_{e,j}\left(j=1\cdots N\right)$. By adding all the discrete pose increments, $L_{arc}$ is calculated as follows:

(15) $$L_{arc}=\sum_{j=1}^{N-1}{∥p_{e,j}-p_{e,j-1}∥}_{2}.$$

In addition to the objectives defined in Equation (14), the trajectory should also satisfy the geometric and electromechanical constraints during the manipulation, including collision avoidance and the limits of joint angles, velocity and acceleration. The valid ranges of these constraints are considered. For the constraint of joint limits, $\Theta_{j}$ , $\Omega_{j}$ and $A_{j}$ at each sampling period are verified in the available work space. Given the joint angles $\Theta_{j}$ at the *j*th sampling moment, the positions of all joints are obtained through forward kinematic mapping Equation (3). The obstacles are simplified and discretized into point set Q. The number of obstacle points in set Q is $N_{Q}$ and each link of the manipulator is simplified as a line segment. We define $d_{min,ij}$ as the shortest distance between the *i*th obstacle in set Q and the *j*th link’s line segment. Once $d_{min,ij}$ is smaller than a predefined safe threshold $d_{safe}$, the aerial manipulator will collide with the obstacle.

Finally, the multi-objective optimization problem for the trajectory planning of aerial grasping is formulated as follows:

(16) $$\begin{array}{cc} \; & \min F\left(B\right)=\{f_{1}\left(B\right),f_{2}\left(B\right)\}\\ \; & s.t.\left\{\begin{array}{cc} \Theta_{i}\in S_{\Theta }, & i=1\cdots N,\\ \Omega_{i}\in S_{\Omega }, & i=1\cdots N,\\ A_{i}\in S_{A}, & i=1\cdots N,\\ Q_{j}\in Q, & j=1\cdots N_{Q},\\ \Theta_{min}\leq \Theta_{i}\leq \Theta_{max}, & i=1\cdots N,\\ \Omega_{min}\leq \Omega_{i}\leq \Omega_{max}, & i=1\cdots N,\\ A_{min}\leq A_{i}\leq A_{max}, & i=1\cdots N,\\ d_{min,ij}>d_{safe}, & i=1\cdots N_{Q},j=1\cdots N,\\ 0<T_{a}\leq t_{max},\\ 0<T_{b}\leq t_{max}, \end{array}\right. \end{array}$$

where $t_{max}$ is the time limit of execution of each quintic curve. $t_{max}$ is set to 5 s empirically in our experiments. $\Theta_{min}$ and $\Theta_{max}$ depend on the specifications of servo motors. $\Omega_{min}$, $\Omega_{max}$, $A_{min}$, and $A_{max}$ depend on both the servo motors and the dynamics of the aerial system.

### 5.3. NSGA-II Based Trajectory Optimization

The study utilizes the well-known optimizer NSGA-II to solve the aforementioned multi-objective optimization problem because NSGA-II is convenient and accurate even with complex constraints. The UAV will be in the hovering mode during grasping. The initial velocities $\Omega_{s}$ are zero and the angles $\Theta_{s}$ are obtained from each servo motor’s sensor. The goal angular velocities $\Omega_{g}$ are set to zero. The goal angles of all joints $\Theta_{g}$ are obtained from the end-effector’s goal position through inverse kinematics. Therefore, the parameter vector is rewritten as follows by removing the known boundary conditions:

(17) $$B_{p}=\left(T_{a},T_{b},A_{s}^{T},\Theta_{m}^{T},\Omega_{m}^{T},A_{m}^{T},A_{e}^{T}\right),$$

where $B_{p}\in R^{k}$, k=5×N+2. The algorithm provides a Pareto solution set [19] in which the first optimization solution is selected as B. Finally, with the solution, the planned trajectory in joint space is calculated using Equations (10) and (12).

## 6. Vision-Based Trajectory Compensation and Tracking

The manipulator and UAV are substantially coupled in dynamics and the coupling can affect the instant poses of the aerial manipulator. Even the pose of the target object can be altered by a few unstable factors, such as wind disturbance. Therefore, following the pre-planned trajectory accurately is challenging for the aerial manipulator. The section proposes a novel and efficient trajectory tracking controller based on kinematic compensation to address the aforementioned problem. Firstly, a visual target tracking controller is developed to guarantee that the target object is within the field of view of the camera. Secondly, the real-time trajectory compensation based on the visual detection is presented. Finally, a trajectory tracking method based on time-differential filtering is given.

### 6.1. Visual Target Tracking

The target object should maintain in the field of view of the camera. It guarantees that the object’s location information is always available for the grasping control. In addition, a simple object tracking controller is then developed as follows by simultaneously controlling the pitch angle of the camera and the UAV yaw angle:

(18) $$\left\{\begin{array}{c} \theta_{out}\left(t\right)=k_{p_{1}}e_{v}\left(t\right)+k_{d_{1}}\Delta e_{v}\left(t\right),\\ \psi_{out}\left(t\right)=k_{p_{2}}e_{u}\left(t\right)+k_{d_{2}}\Delta e_{u}\left(t\right), \end{array}\right.$$

where $\theta_{out}\left(t\right)$ and $\psi_{out}\left(t\right)$ denote the outputs of the camera’s pitch angle and UAV’s yaw angle, respectively, $k_{p_{1}}$, $k_{d_{1}}$, $k_{p_{2}}$, and $k_{d_{2}}$ are constant parameters, and $e_{u}\left(t\right)$, $e_{v}\left(t\right)$, $\Delta e_{u}\left(t\right)$, and $\Delta e_{v}\left(t\right)$ are defined as

(19) $$\left\{\begin{array}{c} e_{u}\left(t\right)=u^{*}-u\left(t\right),\Delta e_{u}\left(t\right)=e_{u}\left(t\right)-e_{u}\left(t-1\right),\\ e_{v}\left(t\right)=v^{*}-v\left(t\right),\Delta e_{v}\left(t\right)=e_{v}\left(t\right)-e_{v}\left(t-1\right), \end{array}\right.$$

where $\left(u^{*},v^{*}\right)$ denotes the expected target position in the image; (u(t),v(t)) denotes the measured target position in the image at time *t*. $\psi_{out}\left(t\right)$ is directly given to the flight autopilot in the UAV’s position control loop [36].

### 6.2. Real-Time Trajectory Compensation

The trajectory planning algorithm in Section 5 provides $T_{e}^{b}\left(t\right)$ designed based on the target object pose $T_{a}^{b}\left(t=0\right)$ at the beginning. However, the movement of manipulator or other unstable factors, such as wind disturbance, may alter the UAV body pose or target object pose during the grasping process. The problem hinders the grasping success if no compensation is introduced. To address the problem, we propose a novel compensation algorithm based on the results of trajectory planning and real-time target object tracking. With the real-time visual tracking system shown in Figure 1a, the UAV continuously tracks the target and provides the value of $T_{a}^{b}\left(t\right)$ in real time. The trajectory planning aims to realize a collision-free target grasping and the body in the grasping process is assumed not to generate a substantial shift. Therefore, only the collision of the end-effector will be considered. The assumption is weak because in all our experiments the UAV generates acceptable movements.

The target object is constantly considered as the position reference, thereby guaranteeing that the transformation $T_{a}^{e}\left(t\right)$ of the target frame relative to the end-effector should remain constant. Let $T_{a}^{b}\left(t\right)$ denote the real-time target tracking result from the camera system. The initial visual observation $T_{a}^{b}\left(0\right)$ and planned trajectory $T_{e}^{b}\left(t\right)$ indicate that we can calculate the trajectory of the target w.r.t. the end-effector as follows:

(20) $$T_{a}^{e}\left(t\right)=T_{b}^{e}\left(t\right)T_{a}^{b}\left(0\right).$$

The new trajectory $T_{er}^{b}$ of the end-effector w.r.t of the UAV body frame is designed as follows by integrating a kinematic compensation

(21) $$T_{er}^{b}\left(t\right)=T_{a}^{b}\left(t\right)T_{e}^{a}\left(t\right).$$

By substituting Equation (20) into Equation (21), we obtain the following equation:

(22) $$\begin{array}{c} T_{er}^{b}\left(t\right)=T_{a}^{b}\left(t\right)T_{b}^{a}\left(0\right)T_{e}^{b}\left(t\right)=T_{a}^{b}\left(t\right)\left[\begin{array}{cc} R_{a}^{bT} & -R_{a}^{bT}t_{a}^{b}\\ 0_{3}^{T} & 1 \end{array}\right]T_{e}^{b}\left(t\right), \end{array}$$

where $R_{a}^{b}$ and $t_{a}^{b}$ denote the rotation and translation parts of $T_{a}^{b}\left(0\right)$ , respectively. Finally, we obtain the compensated joint inputs from $T_{er}^{b}\left(t\right)$ by using the inverse kinematics in Section 4.

### 6.3. Trajectory Tracking Based on Time-Differential Filtering

**Figure 4:**
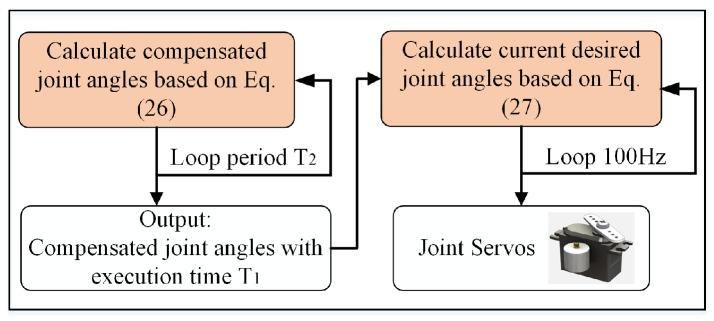
Flow chart of the proposed trajectory following controller.

It is difficult to realize successful aerial grasping by merely applying motion compensation because many unavoidable factors affect the implementation. The target or UAV poses may change after the trajectory planning. The image processing for calculating $T_{a}^{b}\left(t\right)$ causes some time delay and the response time of the servo motors is also limited. The combined effect of these factors will lead to errors between the calculated and required trajectories. Direct use of the calculated trajectory will cause considerable shaking during grasping. The factors inevitably induce challenges for an UAM to follow a trajectory accurately. The study develops an effective filter-based controller to address the problem. The flow chart of the trajectory following controller is illustrated in Figure 4. Once the controller receives the input instruction $\Theta_{et}$, the angle sensor of each servo of the manipulator immediately measures its joint angle. The target joint angles as well as the execution time are given by the following interpolation at a frequency of 100 Hz. Let $t_{st}$ denote the input command timestamp when the target joint angles $\Theta_{et}$ are received from the algorithm of trajectory planning. An execution time $T_{1}$ for the tracking of $\Theta_{et}$ is associated. The joint output at time *t* is provided as follows:

(23) $$\left\{\begin{array}{cc} \Theta \left(t\right)=\Theta_{st}+\frac{\Theta_{et}-\Theta_{st}}{T_{1}}\left(t-t_{st}\right), & t<T_{1}+t_{st},\\ \Theta \left(t\right)=\Theta_{et}, & t\geq T_{1}+t_{st}, \end{array}\right.$$

where $\Theta_{st}$ denotes the angle values at the current time detected by their servo sensors. $T_{2}$ in Figure 4 denotes the period of trajectory compensation. The values of $T_{1}$ and $T_{2}$ depend on the practical specification of the servo motors and are set to 0.2 s and 0.1 s, respectively, in our experiments. The difference between the execution time $T_{1}$ and compensation period $T_{2}$ will provide a filtering effect on the trajectory tracking, thereby addressing the considerable shaking problem during grasping.

## 7. Experiments and Discussion

The electric hardware system of the proposed aerial manipulator mainly comprises a pixhawk flight controller, servo motor controller for the manipulator joints and grippers and an onboard computer Intel NUC i7-5557U. The computer runs Ubuntu14.04 with ROS-indigo. The target tracking camera is a low-cost complementary metal oxide semiconductor (CMOS) camera with a resolution of 640×480 at 60 Hz.

Because the computer vision research on object tracking is beyond the paper’s scope, the QR-code Apriltag [35] was attached to the target for simplicity; the tag provides the 6-DoF relative target pose w.r.t. the camera frame. The binocular or RGB-D camera can also be used to provide the 6-DoF pose information. The pose detection frequency of an Apriltag reached an average of 23 Hz on our onboard computer. Because UAV’s pose estimation (i.e., localization or simultaneous localization and mapping (SLAM)) [37]) is beyond the paper’s scope, we utilized the motion capture system for the pose feedback of the UAV control. In addition, the grasping process is completely autonomous with an onboard computer and sensors. The wheelbase and payload of the octocopter are 55 cm and 4.0 Kg, respectively. The total weight of the system is 5.45 Kg and the arm weight is 0.545 Kg. The maximum thrust capability is approximately 9 Kg and the payload capability of the manipulator is approximately 0.2 Kg.

### 7.1. Verification of Multi-Objective Optimization-Based Trajectory Planning

**Figure 5:**
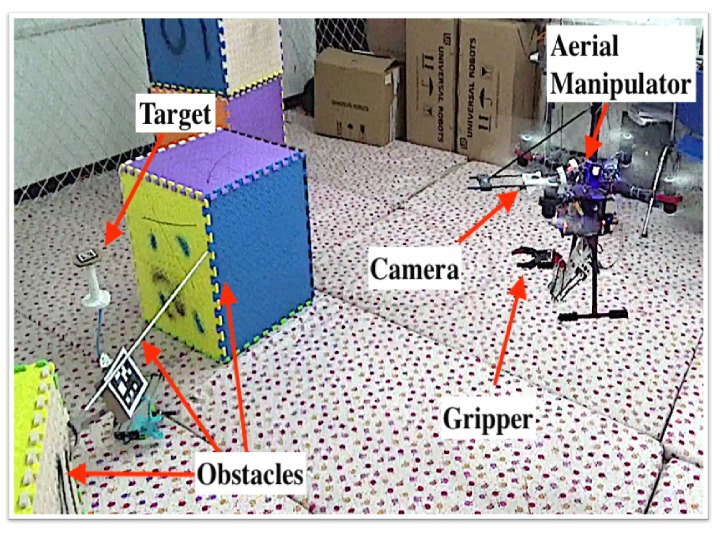
Manipulator and experimental object to be grasped.

An example was performed to verify the multi-objective optimization-based trajectory planning method. The parameters of NSGA-II for the experiments were set as follows: populations as 60, mutation rate as 0.5, iterations as 150, crossover distribution index as 50, crossover rate as 1.0, and mutation distribution index as 20. The experimental object to be grasped is illustrated in Figure 5. The target object is shaped similar to a mushroom and can only grasp along the horizontal direction. Trajectory planning should guarantee safe grasping. The initial and target angles of the wrist joint of the end-effector were set to zero, that is, maintained horizontally. The velocity and acceleration limit for each joint were set to [-π/2,π/2] rad/s and [-π,π] rad/s${}^{2}$, respectively. Each single-stage maximum duration was set to 5 s, that is, $T_{a}\in \left(0,5\right]$ and $T_{b}\in \left(0,5\right]$.

**Figure 6:**
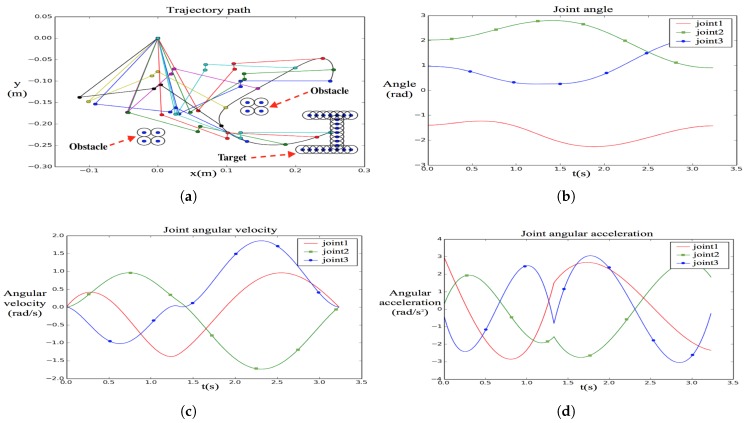
Planning results of our proposed trajectory planning algorithm. (**a**) Trajectory path; (**b**) joint angle; (**c**) joint angular velocity; (**d**) joint angular acceleration.

Table 2 illustrates the solution in the front of the Pareto [19] obtained by the proposed planning algorithm. Two obstacles were set in the environment. The time cost of the trajectory planning is 2.460 s. The planned trajectory and curves of each joint’s angle, velocity and acceleration are shown in Figure 6. The trajectory diagram shows the obstacle point cloud with a safe distance and the collision-free trajectory of the aerial manipulator. All physical variables satisfied the limitation constraints, and the accelerations were smooth. The accelerations were continuous and smooth in nearly all parts.

### 7.2. Experiments of Trajectory Following

**Figure 7:**
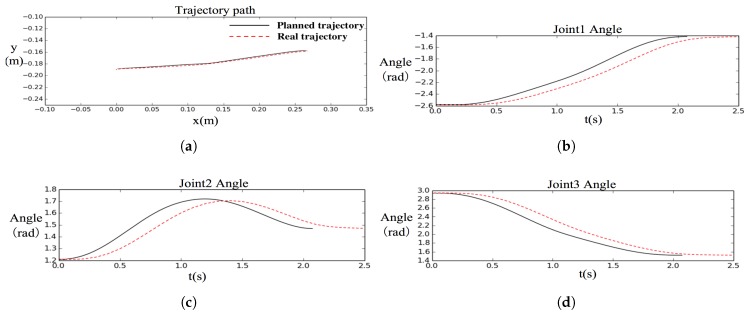
Tracking result of the proposed time-differential filtering-based controller. (**a**) Trajectory path; (**b**) joint1 angle; (**c**) joint2 angle; (**d**) joint3 angle.

Another experiment was performed on the aerial manipulator to verify the trajectory following controller in Section 6. The parameters of controllers $T_{1}$ and $T_{2}$ were set to 0.2 s and 0.1 s, respectively. In the experiment, we should verify that the filtering-based controller can control the end-effector to track the desired trajectory. For convenience, the aerial manipulator was tested on the ground. The end-effector’s trajectory was generated from (0.0, −0.19) to (0.27, −0.16), which is represented by the black curve in Figure 7. The red and dotted curve represents the tracking result detected from the sensor output of each joint servo. From Figure 7, it is seen that the trajectory following controller played a smooth tracking. The tracking results on the joints delayed approximately 0.2 s compared with the desired trajectory because of the filtering effect in the proposed controller. The controller has substantially reduced the tracking joggles and the real trajectory still coincided with the desired one. Experiments on aerial grasping were further performed to verify that the delays do not affect the grasping performance in the following subsection.

### 7.3. Experiment Results of the Aerial Grasping

**Figure 8:**
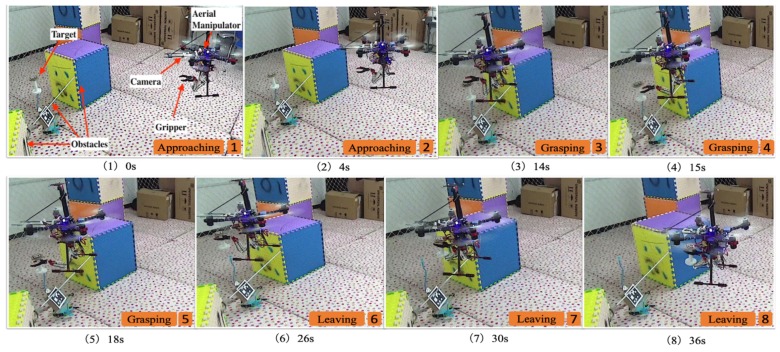
Snapshots of the aerial grasping.

The global localization of UAV was provided by the motion capture system for system safety because the UAV autopilot system is not the focus of the study. The grasping task in the experiment is divided into two phases. Firstly, the aerial manipulator flies towards the target object by using the onboard visual tracking system and remains in hovering mode when a place with sufficient grasping working space is reached. Secondly, the aerial manipulator performs the grasping of the target object. Snapshots of the aerial grasping procedure are presented in Figure 8. During the experiment, the position of the aerial manipulator and target object shook with a maximum magnitude of approximately 8 cm because of the dynamic instability and wind interference. Nevertheless, our proposed approach still achieved a good performance. The performance is evidently observed in the attached video of the experiment. Several experiments were performed by changing the hovering position of the UAV, where the object was grasped successfully. (We provided an experiment video as an Supplementary Material to illustrate the performance of our approach).

## 8. Conclusions

The paper proposes a novel approach for autonomous grasping with a multi-DoF lightweight aerial manipulator. A lightweight manipulator is firstly designed to reduce the dynamic interference to the system. The UAV is equipped with a monocular camera system to provide the target object’s location information. To implement autonomous grasping, a framework based on the visual information is developed, comprising visual target tracking, trajectory generation without collision and trajectory tracking control. The trajectory planning for aerial grasping control is formulated as a multi-objective optimization problem, whilst motion constraints and collision avoidance are considered in the optimization. The NSGA-II is applied to determine the optimal solution. A vision-based trajectory compensation and tracking control method is further introduced to address the external disturbance and the coupled affection between the manipulator and octocopter. Finally, several experiments are performed to illustrate the effectiveness of the proposed approach. The current work focuses only on the grasping process, and there are still many challenging problems to be addressed in the field of aerial manipulation and transportation. To make the aerial manipulator usable in practical applications, our future work will include the completely autonomous ability in complex environments, mainly focusing on the state estimation, obstacle detection, and force control.

## Figures and Tables

**Table 1:** Denavit–Hartenberg parameters.

Link *i*	$\alpha_{i}$ (rad)	$a_{i}$ (mm)	$d_{i}$ (mm)	$\theta_{i}$ (rad)
1	0	179	0	$\theta_{1}$
2	0	110	0	$\theta_{2}$
3	π/2	13	0	$\theta_{3}$
4(e)	0	0	130	$\theta_{4}$

**Table 2:** Optimal trajectory planning result 2.

Objective 1 (s)		Objective 2 (m)		$T_{a}$ (s)	$T_{b}$ (s)
3.22954		0.412345		1.33109	1.89846
**Joint**	$A_{s}$	$\Theta_{m}$	$\Omega_{m}$	$A_{m}$	$A_{e}$
1	2.98581	−1.8748	−1.3166	1.50605	−2.3504
2	0.23981	2.78616	0.16251	−1.5779	1.82654
3	−0.3575	0.25308	0.02344	−0.8158	−0.2502

## Supplementary Materials

The following are available online at https://www.mdpi.com/1424-8220/19/19/4253/s1.

## Abbreviations

The following abbreviations are used in this manuscript:

UAV	Unmanned Aerial Vehicle
UAM	Unmanned Aerial Manipulator
DoF	Degree of Freedom
NSB	Null Space-based Behavioral
DMPs	Dynamic Movement Primitives
PDMPs	Parametric Dynamic Movement Primitives
RRT	Rapidly exploring Random Tree
MPC	Model Predictive Control
